# Structural and functional characterization of the nucleotide-binding domains of ABCA4 and their role in Stargardt disease

**DOI:** 10.1016/j.jbc.2024.107666

**Published:** 2024-08-14

**Authors:** Jessica Fernandes Scortecci, Fabian A. Garces, Jai K. Mahto, Laurie L. Molday, Filip Van Petegem, Robert S. Molday

**Affiliations:** Department of Biochemistry & Molecular Biology, University of British Columbia, Vancouver, British Columbia, Canada

**Keywords:** ABC transporters, ABCA4 structure, cryoelectron microscopy, Stargardt disease, Walker A and B motifs, nucleotide-binding domains, missense mutations, ATPase activity

## Abstract

ABCA4 is an ATP-binding cassette (ABC) transporter that prevents the buildup of toxic retinoid compounds by facilitating the transport of *N*-retinylidene-phosphatidylethanolamine across membranes of rod and cone photoreceptor cells. Over 1500 missense mutations in ABCA4, many in the nucleotide-binding domains (NBDs), have been genetically linked to Stargardt disease. Here, we show by cryo-EM that ABCA4 is converted from an open outward conformation to a closed conformation upon the binding of adenylyl-imidodiphosphate. Structural information and biochemical studies were used to further define the role of the NBDs in the functional properties of ABCA4 and the mechanisms by which mutations lead to the loss in activity. We show that ATPase activity in both NBDs is required for the functional activity of ABCA4. Mutations in Walker A asparagine residues cause a severe reduction in substrate-activated ATPase activity due to the loss in polar interactions with residues within the D-loops of the opposing NBD. The structural basis for how disease mutations in other NBD residues, including the R1108C, R2077W, R2107H, and L2027F, affect the structure and function of ABCA4 is described. Collectively, our studies provide insight into the structure and function of ABCA4 and mechanisms underlying Stargardt disease.

ABCA4 is a member of the A-subfamily of ATP-binding cassette (ABC) transporters which is primarily expressed in vertebrate rod and cone photoreceptors. It uses the energy from ATP hydrolysis to transport *N*-retinylidene-phosphatidylethanolamine (*N*-Ret-PE), the Schiff-base adduct of retinal and phosphatidylethanolamine (PE), across photoreceptor outer segment disc membranes (1, 2, 3, 4, 5). This prevents the buildup of toxic retinoid compounds by facilitating the removal of *all-trans* retinal (ATR) that is produced through photoexcitation and excess *11-cis* retinal not required for the regeneration of rhodopsin or cone opsins (6, 7).

The importance of ABCA4 in photoreceptor physiology is evident from the finding that over 2,300 mutations in the *ABCA4* gene have been implicated in Stargardt disease (STGD1) and related cone-rod dystrophy (https://databases.lovd.nl/shared/variants/ABCA4/). STGD1 is an autosomal recessive retinal degenerative disease characterized by the accumulation of fluorescent flecks in the central retina, atrophy of the retinal pigment epithelium (RPE), and progressive degeneration of rod and cone photoreceptors (8, 9, 10). Affected individuals typically lose central vision in their first or second decade of life, although in some cases, the disease is manifested much later in life due to residual transport activity of some ABCA4 variants (11, 12, 13). In the absence or reduction of ABCA4 transport activity, *N*-Ret-PE and retinal accumulate in disc membranes where they react to form di-retinoid compounds including A2PE, the pyridinium di-retinoid formed from the condensation of one molecule of retinal (vitamin A aldehyde) and one molecule of *N*-Ret-PE (14, 15). Upon phagocytosis of photoreceptor outer segments by RPE cells, phosphatidic acid is removed from A2PE by phospholipase D to form A2E (a compound made up of two molecules of retinal and one molecule of ethanolamine). A2E and other di-retinoids accumulate as fluorescent lipofuscin deposits in RPE cells of STGD1 patients and *Abca4* KO and transgenic mice (16, 17, 18, 19, 20, 21). STGD1 affects as many as 1 in 6,500 individuals (22) and therefore is a prominent target for drug and gene therapeutic interventions although to date there are no treatments for this disease (23, 24).

The human *ABCA4* gene consisting of 50 exons encodes a 2273 amino acid full transporter, previously known as the Rim protein (8, 25). It is organized as two nonequivalent tandem halves, with each half containing a transmembrane domain (TMD) consisting of six membrane spanning segments, a nucleotide-binding domain (NBD), and a large glycosylated exocytoplasmic domain (ECD) (26). Recently, the structures of ABCA4 in various states have been determined by cryo-EM (27, 28, 29). These studies have shown that ABCA4 in its apo state and *N*-Ret-PE substrate-bound state exists in an open outward conformation with the high-affinity binding site for *N*-Ret-PE located between the two TMDs and ECD1 and accessible through lateral diffusion of *N*-Ret-PE from the lumen leaflet of disc membranes. In its nucleotide-bound state, an ATPase-deficient ABCA4 variant exhibits a closed conformation with the two ATP-Mg^2+^ complexes sandwiched between the two NBDs and the two TMDs in close contact with each other. Two regulatory domains (RDs), RD1 and RD2, first described in the related transporter ABCA1 (30), are present just downstream of NBD1 and NBD2 and together with pinning helices appear to play an important role in maintaining the NBDs in close contact with each other during the reaction cycle (27).

Although considerable progress has been made on the structural and functional characterization of ABCA4 (7), the role of the individual NBDs in context to the full transporter has not been clearly defined. Both NBDs contain the characteristic features found in other ABC transporters including the Walker A, Walker B, and signature motifs and the A, D, H, and Q loops important for ATP binding and hydrolysis (31, 32, 33). When expressed in *Escherichia coli*, each NBD has been reported to display ATPase activity (34, 35). Photoaffinity labeling of full-length ABCA4 revealed differences in the reactivity of the NBDs to an azido-ATP analogue (36).

Some ABC transporters require ATP binding and hydrolysis at both nucleotide-binding sites, while others contain one degenerate nucleotide-binding site that enables ATP binding, but not hydrolysis (33). Furthermore, the structures of ABC proteins with Glu to Gln (E-Q) mutations in the Walker B motif containing bound ATP do not always resemble the structures of the WT protein containing nonhydrolyzable ATP analogues (17, 31). In this study, we have determined the structure of WT ABCA4 containing the nonhydrolyzable ATP analog adenylyl-imidodiphosphate (AMP-PNP) by cryo-EM for comparison with the previously published structure of E-Q double mutant in its ATP-bound state. We have also defined the role of conserved residues in the Walker A and Walker B motifs of individual NBDs on the ATPase activity and substrate binding properties of ABCA4. Finally, we have investigated the effect on a number of disease-associated missense mutations in the NBDs on the expression, activity, and structure of ABCA4. Our studies indicate that ATPase activity in both nucleotide-binding sites is required for a functionally active transporter. We have used the structures of ABCA4 and functional properties to define molecular mechanisms underlying STGD1 linked to mutations in the NBDs. These results are relevant to understanding the structure–function relationships of other ABCA transporters linked to human disorders (37, 38) and serve as a framework for developing rationale based therapeutic treatments for STGD1.

## Results

### Structure of ABCA4 containing bound AMP-PNP

**Figure 1:**
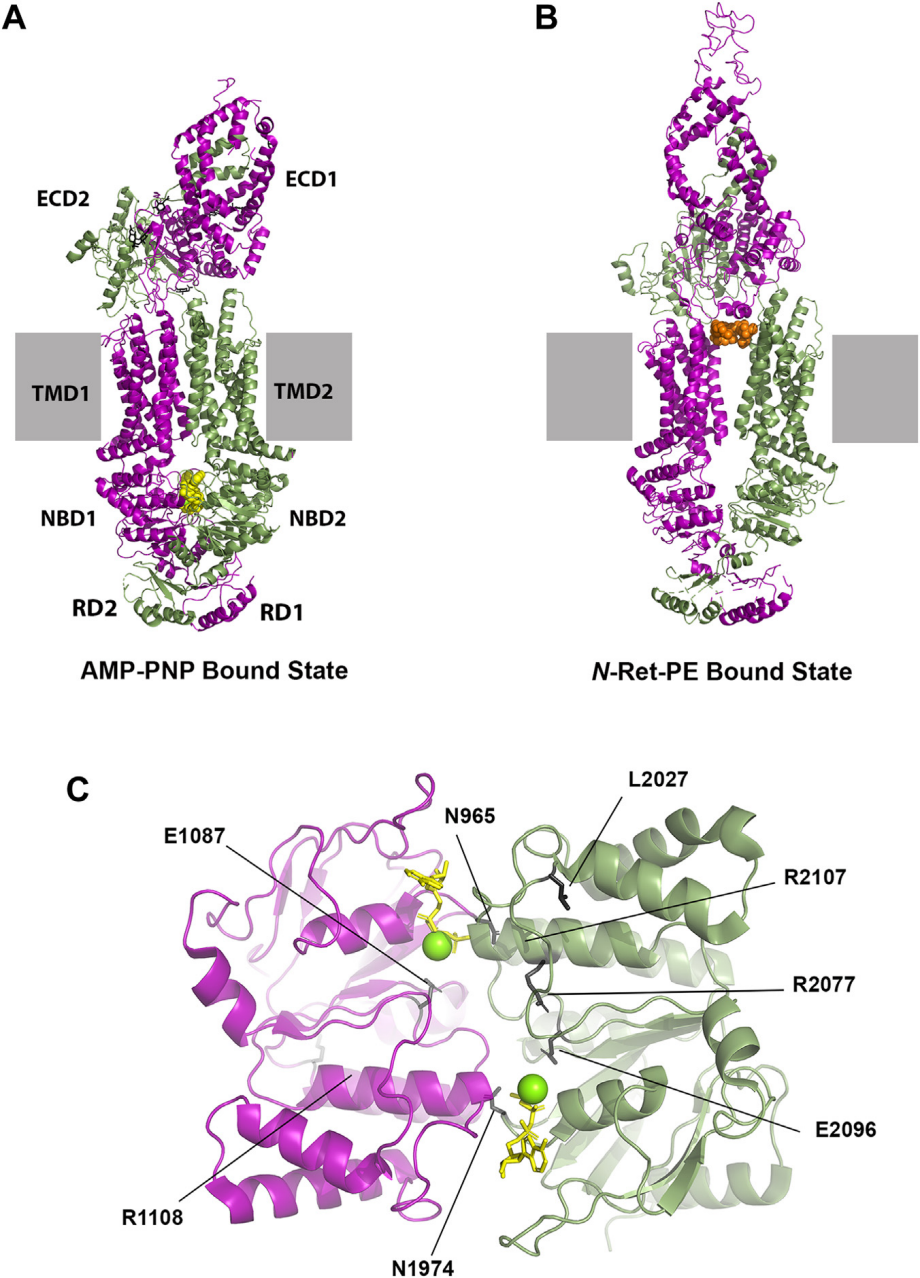
**Structures of ABCA4 in the nucleotide and substrate-bound states.***A*, structure of ABCA4 in the nucleotide (AMP-PNP) bound, closed state (PDB ID: 8F5B). The N-half and C-half are colored in *purple* and *green*, respectively. AMP-PNP (*yellow spheres*) are sandwiched between the NBDs. ECD, exoplasmic domain; NBD, nucleotide-binding domain; RD, regulatory domain; TMD, transmembrane domain. *Black sticks* represent oligosaccharide chains. *B*, substrate bound, open outward state (PDB ID: 7M1Q) shown for comparison with the *N*-Ret-PE substrate as *orange spheres*. *C*, NBDs of ABCA4 in its AMP-PNP bound state. AMP-PNP shown as *sticks* and Mg^2+^ ions shown as *spheres*. The location of key residues investigated in this study and associated with STGD1 are shown in *black sticks*. ABC, ATP-binding cassette; *N*-Ret-PE, *N*-retinylidene-phosphatidylethanolamine; PDB, Protein Data Bank; PE, phosphatidylethanolamine; STGD1, Stargardt disease.

**Figure 2:**
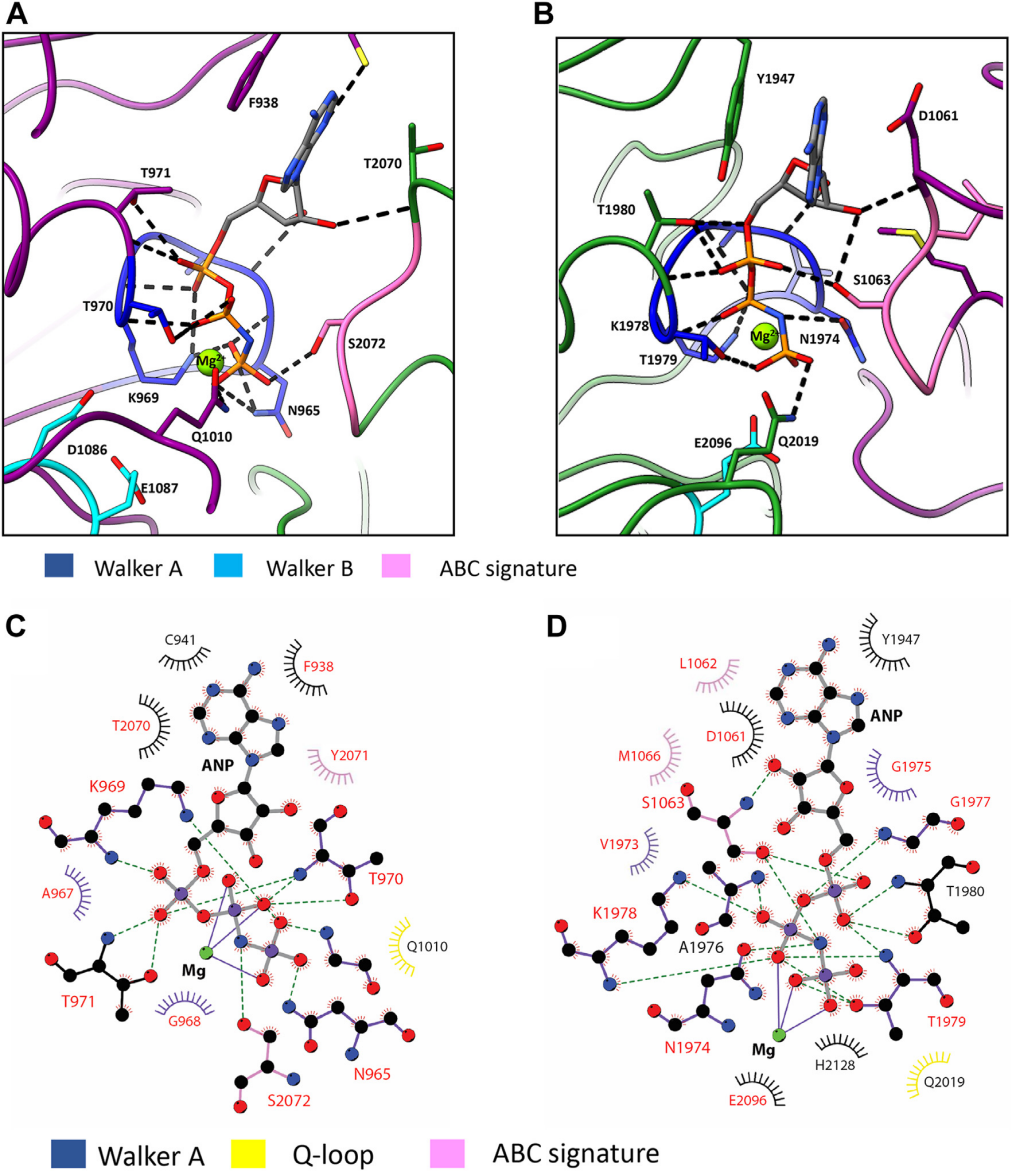
**Structure and interaction of AMP-PNP (ANP) with residues within NBD1 and NBD2.***A*, interaction of ANP with residues from Walker A motif (*blue*) of NBD1 and ABC signature motif (*pink*) of NBD2 for ATPase site 1. NBD1 is shown in *purple* and NBD2 is in *green*. *B*, interactions of ANP with residues from Walker A motif (*blue*) of NBD2 and signature motif of NBD1 for ATPase site 2. Polar contacts are highlighted in *dashed gray lines*. *C*-*D*, LigPlot+ (57) showing main contacts (within 4 Å) between ANP and residues within the NBDs for ATPase site 1 and ATPase site 2 of WT ABCA4 (PDB 8F5B). *Arcs* indicate van der Waals interacting residues. *Dashed lines* represent H-bonds, while *solid lines* denote coordination bonds. Residues associated with disease-causing missense mutations are labeled in *red*. ABC, ATP-binding cassette; NBD, nucleotide-binding domain; PDB, Protein Data Bank.

To gain further insight into ABCA4 in its nucleotide-bound state, we determined the structure of WT ABCA4 containing the bound nucleotide-derivative AMP-PNP in both nucleotide-binding sites at an average resolution of 3.9 Å by cryo-EM (Fig. 1*A*). In this nucleotide-bound state, ABCA4 displayed a closed conformation distinct from the outward-facing open conformation previously observed for the apo and *N*-Ret-PE bound states (Fig. 1*B*). The two TMDs are in close contact with each other over the entire length of the membrane resulting in the collapse of the cavity for *N*-Ret-PE substrate binding. This agrees with earlier biochemical studies showing that the addition of either AMP-PNP or ATP causes a loss in *N*-Ret-PE binding to ABCA4 (39). The ECDs remain entwined in the nucleotide-bound state, but are now twisted and angled more toward the membrane. The two AMP-PNP molecules, each coordinated with Mg^+2^, are sandwiched between the NBDs arranged as a head-to-tail dimer (Fig. 1*C*). The phosphate groups of AMP-PNP come in close contact with residues from the Walker A motifs and Q-loop of one NBD and residues within the ABC signature motif of the opposing NBD (Fig. 2, *A* and *B*). The adenine group of AMP-PNP is stabilized through π-stacking with residues F938 and Y1947 of the A-loop in NBD1 and NBD2 of ATPase site 1 and 2, respectively, as previously observed for the ABCA4 EQ double mutant (E1087Q/E2096Q) (27, 29). Other interactions between AMP-PNP and residues within the two ATP binding sites are shown in Figure 2, *C* and *D*. Overall the structure of WT ABCA4 with bound AMP-PNP is highly similar to the ABCA4-EQ double mutant containing bound ATP (Protein Data Bank ID 7E7Q) with an RMSD = 1.19 Å for 1645 Cα atoms.

### Effect of mutations in the Walker B glutamate residues on the expression and ATPase activity of ABCA4

**Figure 3:**
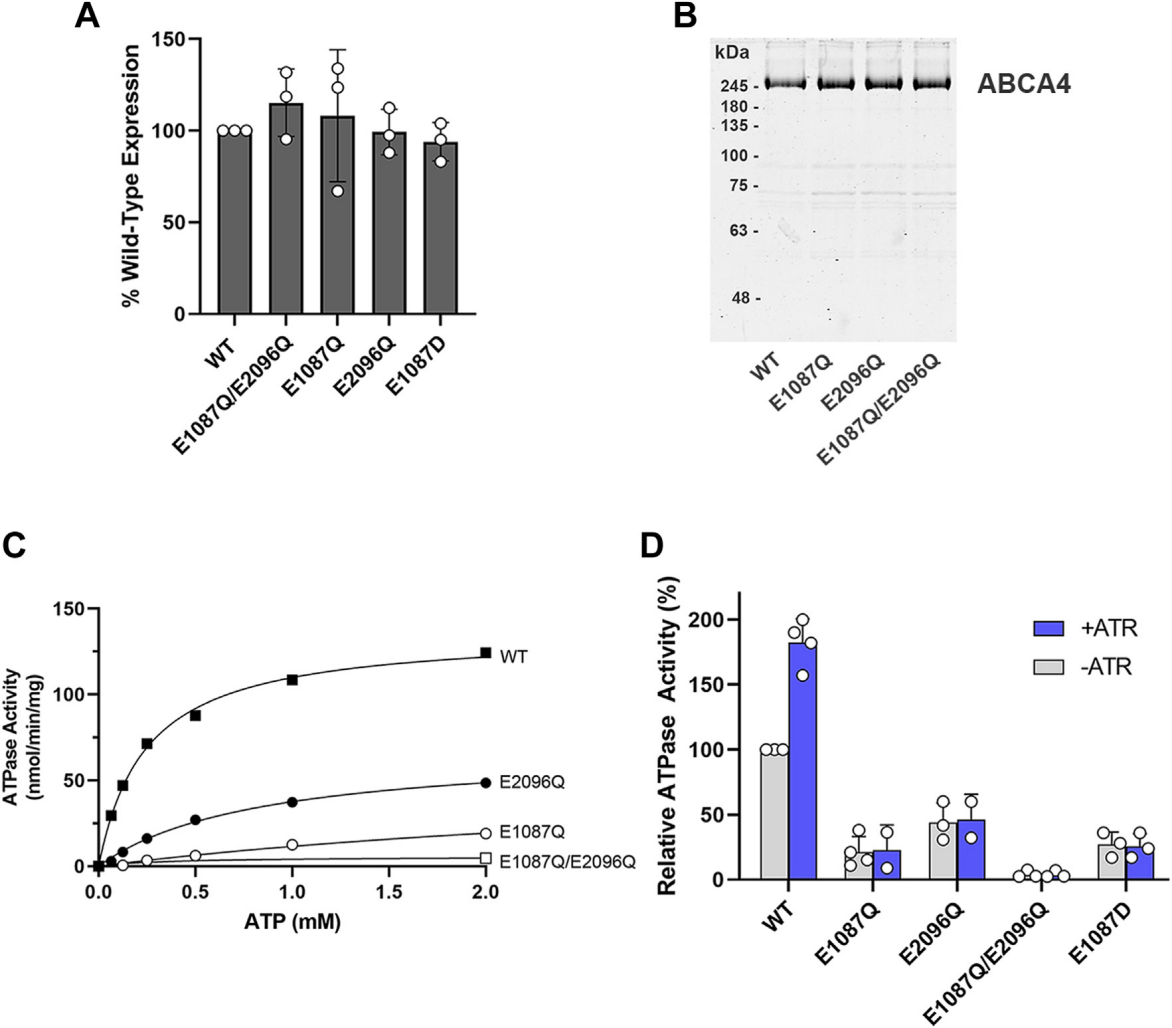
**Expression and ATPase activity of ABCA4 variants with mutations in the glutamate residue of the Walker B motif.***A*, expression level of ABCA4 E-Q variants isolated from transfected HEK293T cells relative to WT ABCA4. Cells expressing ABCA4 variants were solubilized in CHAPS detergent and analyzed on Western blot after centrifugation to remove any aggregated protein. Data are the mean ± SD for three independent experiments. *B*, representative Coomassie blue stained gel showing affinity purified WT and the E-Q variants. *C*, specific ATPase activity of immunoaffinity purified ABCA4 variants as a function of increasing ATP concentration. Curve using the Michael–Menten relationship yielded a V_max_ of 136 nmoles/min/mg protein and a K_m_ of 0.24 mM ATP for WT ABCA4; a V_max_ of 68 nmoles/min/mg protein and K_m_ of 0.83 mM ATP for E2096Q; a V_max_ of 58 E1097Q and K_m_ of 3.96 mM ATP for E1087Q with more limited ATP hydrolysis activity for the latter. *D*, percent ATPase activity relative to WT ABCA4 in the absence of all-*trans* retinal (ATR) at 1 mM ATP. WT ABCA4 displays a 2-fold increase in activity in the presence of 40 μM ATR used to generate *N*-Ret-PE substrate. The basal activity of the variants was significantly reduced and the substrate-activated activity was abolished. Data are mean ± SD for independent experiments represented by data points (*circles*). ABC, ATP-binding cassette; ATR, all-*trans* retinal; *N*-Ret-PE, *N*-retinylidene-phosphatidylethanolamine; PE, phosphatidylethanolamine.

The glutamate residue (E) in the Walker B motif (hhhhDE, where h is a hydrophobic residue) is not directly involved in ATP binding, but is required for ATP hydrolysis (31). When both Walker B glutamate residues are replaced with glutamine (E1087Q/E2096Q referred to as the EQ double mutant), ABCA4 binds two molecules of ATP but is devoid of ATP hydrolysis (27, 29). To assess the contribution of each NBD on the basal and substrate-activated ATPase activities of ABCA4, we expressed the single mutants (E1087Q and E2096Q) for comparison with WT ABCA4 and the double EQ variant. As shown in Figure 3*A*, the single mutants like the double mutant express at levels similar to WT ABCA4 indicating that these substitutions do not adversely affect global protein folding. The E1087D variant implicated in STGD1 (40) also expressed at WT levels.

In order to determine the effect of these mutations on the ATPase activities, the ABCA4 variants were first purified on an immunoaffinity matrix (Fig. 3*B*). The basal ATPase activity of WT ABCA4 exhibited Michaelis–Menten kinetics while the EQ double mutant was devoid of activity as previously reported (27, 28, 29). The ATPase activities of the single ABCA4 EQ variants were significantly reduced relative to WT ABCA4 (Fig. 3, *C* and *D*). The E1087Q variant displayed an ATPase activity which was 21% that of WT ABCA4 at 1 mM ATP concentration while the activity of the E2096Q variant had a higher activity of about 44% WT level. However, unlike WT ABCA4, neither the E1087Q nor the E2096Q variant displayed substrate-stimulated ATPase activity when assayed in the presence of ATR and PE used to generate the *N*-Ret-PE substrate (Fig. 3*D*). The corresponding E1087D variant linked to STGD1 also showed low basal ATPase activity that lacked stimulation by *N*-Ret-PE. These results indicate that glutamate residues in both Walker B motifs are required for substrate stimulated ATPase activity of ABCA4 and imply that this activity is required for the function of ABCA4 as a retinoid transporter.

### The effect of asparagine substitutions in the NBD1 and NBD2 Walker A motifs on the expression and functional activity of ABCA4

The Walker A motifs in NBD1 and NBD2 of ABCA4 each contain an asparagine residue (N965 in NBD1 (GHNGAGKT) and N1974 in NBD2 (GVNGAGKT)). These residues are highly conserved in other ABCA family members (Fig. S1). Substitutions of these asparagine residues with other amino acids (N965D/K/S/Y and N1974S) have been genetically linked to STGD1 (https://databases.lovd.nl/shared/variants/ABCA4/unique). The N965S variant was previously studied and shown to have reduced substrate-stimulated ATPase activity when expressed in HEK293T cells (13). This correlated with the moderate to severe phenotype displayed by STGD1 patients homozygous for this mutation (41).

We have now determined the effect of replacing N965 and N1974 with other residues, (aspartate (D), lysine (K), alanine (A), tyrosine (Y), and glutamine (Q)) on the expression and subcellular localization of ABCA4 in transfected HEK293T cells. After solubilization in CHAPS detergent and removal of any aggregated protein by centrifugation, these ABCA4 variants expressed at levels comparable to WT ABCA4 (Fig. S2). Furthermore, immunofluorescence microscopy of HEK293T cells overexpressing these variants displayed vesicle-like structures together with a more limited reticular distribution as found for WT ABCA4 suggesting that a significant fraction of these variants was able to fold in a native-like conformation and exit the endoplasmic reticulum (Fig. S3).

**Figure 4:**
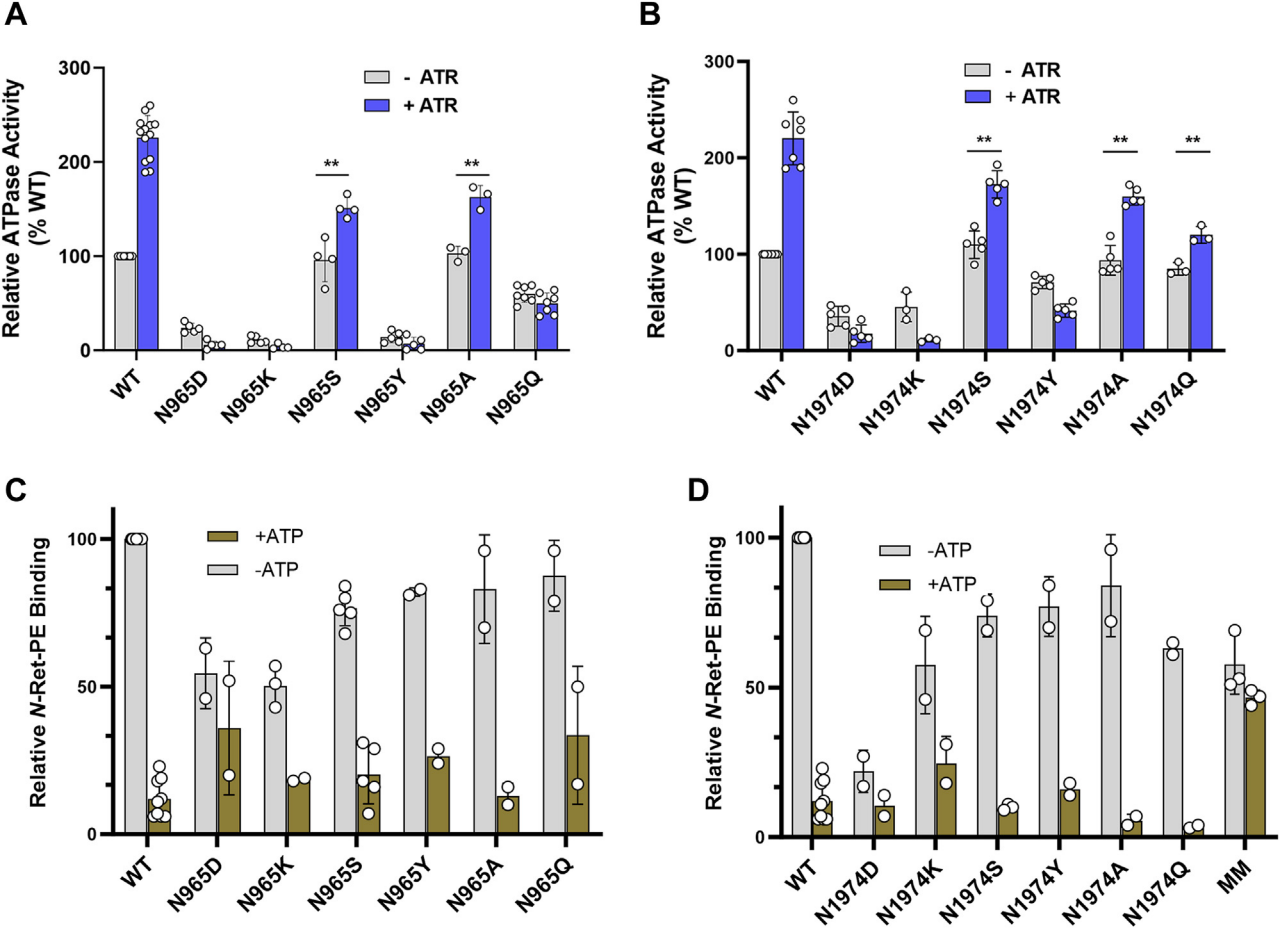
**ATPase activity and *N*-retinylidene-phosphatidylethanolamine (*N*-Ret-PE) binding of ABCA4 variant harboring mutations in the asparagine residues of the NBD1 and NBD2 Walker A motifs.***A*, basal (−ATR) and substrate-activated (+ATR) activity relative to basal WT ATPase activity for N965 variants. Data are the mean ± SD for the number of independent experiments shown by data points. *B*, basal and substrate-activated activity relative to basal WT activity for N1974 variants. Data are the mean ± SD for the number of independent experiments shown by data points. *C*, binding of *N*-Ret-PE to ABCA4 N965 variants in the absence and presence of 2 mM ATP relative to WT ABCA4 in the absence of ATP. *D*, binding of *N*-Ret-PE to ABCA4 N1974 variants in the absence and presence of 2 mM ATP relative to WT ABCA4 in the absence of ATP. Data are mean ± SD for 2 or more independent experiments in both C and D. P values ≤ 0.01 ∗∗. ABC, ATP-binding cassette; ATR, all-*trans* retinal; NBD, nucleotide-binding domain; *N*-Ret-PE, *N*-retinylidene-phosphatidylethanolamine; PE, phosphatidylethanolamine.

To determine the ATPase activity, WT and ABCA4 N965 and N1974 variants were purified by affinity chromatography in the presence of PE (Fig. S4). The ATPase activity was then measured in the presence and absence of ATR. As shown in Figure 4, *A* and *B*, the basal and *N*-Ret-PE stimulated ATPase activities varied for the different mutants. Substitutions of the asparagine residues in the NBD1 Walker A motif generally showed a similar profile as analogous substitutions in the NBD2 Walker A motif. Aspartate, lysine, and tyrosine substitutions resulted in a severe reduction of basal ATPase activity and the absence of *N*-Ret-PE stimulated ATPase activity. The alanine and serine substitutions both retained significant basal ATPase levels, but showed reduced substrate stimulated activity compared to WT. The glutamine substitutions showed some differences with a loss in substrate-stimulated activity for the N965Q variant and a small degree of substrate activated activity for N1974Q variant. For a few variants, the addition of ATR resulted in a small decrease in ATPase activity. This has been previously reported (42) and may be due to nonspecific effects of retinoid binding to partially misfolded variants.

Previously, we showed that the addition of ATP to purified WT ABCA4 containing bound *N*-Ret-PE resulted in the loss in substrate binding (43). This is consistent with the conformational change from an open conformation in the absence of ATP to a closed conformation upon the addition of ATP resulting in a loss in *N*-Ret-PE binding. We have now investigated the effect of the replacement of N965 and N1974 with other residues on *N*-Ret-PE binding and its release by ATP. Figure 4, *C* and *D* shows that all the variants were able to bind *N*-Ret-PE in the absence of ATP. However, the extent of substrate binding varied such that the S, Y, and A substitutions retained 75 to 88% of binding while the D, K, and Q substitutions showed 50 to 63% *N*-Ret-PE binding except for the N1974D variant which showed only 25% binding. Substrate binding was effectively reduced upon the addition of 1 mM ATP in all cases. These results indicate that substitution of the asparagine in the Walker A motifs with other residues moderately impair the binding of substrate, most likely due to long-range coupling between the NBDs and the substrate-binding site. The release of the substrate upon the addition of ATP suggests that the NBDs are capable of binding ATP and initiating a conformational change to the closed state of ABCA4 leading to the loss in *N*-Ret-PE binding. The MM double mutant in which the lysine residues in both Walker A motifs were substituted with methionine showed a modest reduction in *N*-Ret-PE binding which was not significantly reduced upon the addition of 1 mM ATP. The inability of ATP to release *N*-Ret-PE in the MM variant is likely due to the loss or significant decrease in ATP binding of this variant thereby preventing the transition to the closed state of ABCA4.

### Reassessment of ATPase activity of ABCA4-N965S transgenic mice

Transgenic mice harboring a N965S mutation in ABCA4 were previously reported to display a decrease in ABCA4 expression and trafficking to the outer segments of rod photoreceptor cells (21). Furthermore, the ATPase activity of the affinity purified N965S ABCA4 protein after reconstitution into liposomes lacked ATR-stimulated ATPase activity. This latter result is in contrast to detergent-solubilized N965S ABCA4 variant isolated from transfected HEK293T cells (13) and reproduced in Figure 4*A*.

**Figure 5:**
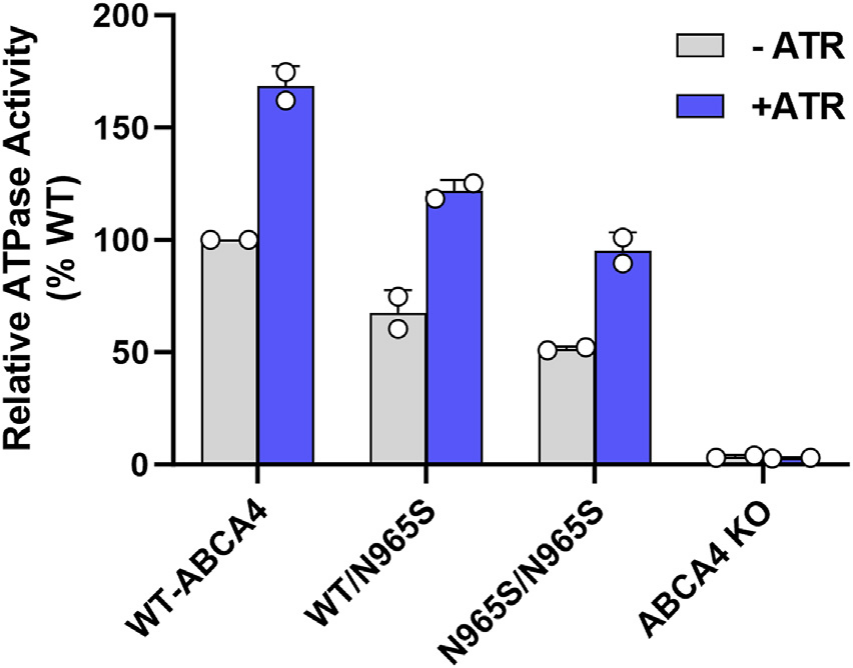
**ATPase activity of ABCA4 variants from transgenic and KO mice.** Retinal extracts from WT mice, N965S heterozygous mice, N965S from homozygous mice, and ABCA4 KO mice were solubilized in CHAPS. ABCA4 variants were purified on an affinity column and their ATPase activity was measured in the absence and presence of 40 μM all-*trans* retinal (ATR) in buffer containing phosphatidylethanolamine lipid. Data are the average for two independent experiments. ABC, ATP-binding cassette.

We surmise that the difference in activities of the N965S variants isolated from mouse photoreceptors and HEK293 cells may have arisen from the sample preparation used in the ATPase assays and not a difference between mouse and human ABCA4 variants or the type of cells expressing the protein. More specifically, the ATPase activity of the N965S variant from mouse photoreceptors was determined after reconstitution of the ABCA4 variant into liposomes, whereas the activity of the N965S variant expressed and purified from HEK293T was assayed directly in detergent solution. To test this, we have now measured the ATPase activity of the detergent-solubilized ABCA4 N965S variant purified from N965S transgenic mice together with samples from WT and ABCA4 KO mice. Figure 5 shows that the N965S ABCA4 variant isolated from N965S homozygous and heterozygous mice exhibited both basal and ATR-activated ATPase activity when measured in the presence of detergent although at a reduced level relative to WT ABCA4 in general agreement with the human recombinant protein from transfected HEK293T cells. This suggests that the purified N965S variant from transgenic mice did not reconstitute effectively into lipid vesicles as an active protein in the earlier studies.

### Walker A asparagine residues forms polar contacts with the nucleotide γ-phosphate and the aspartic acid of the opposing D-loop

**Figure 6:**
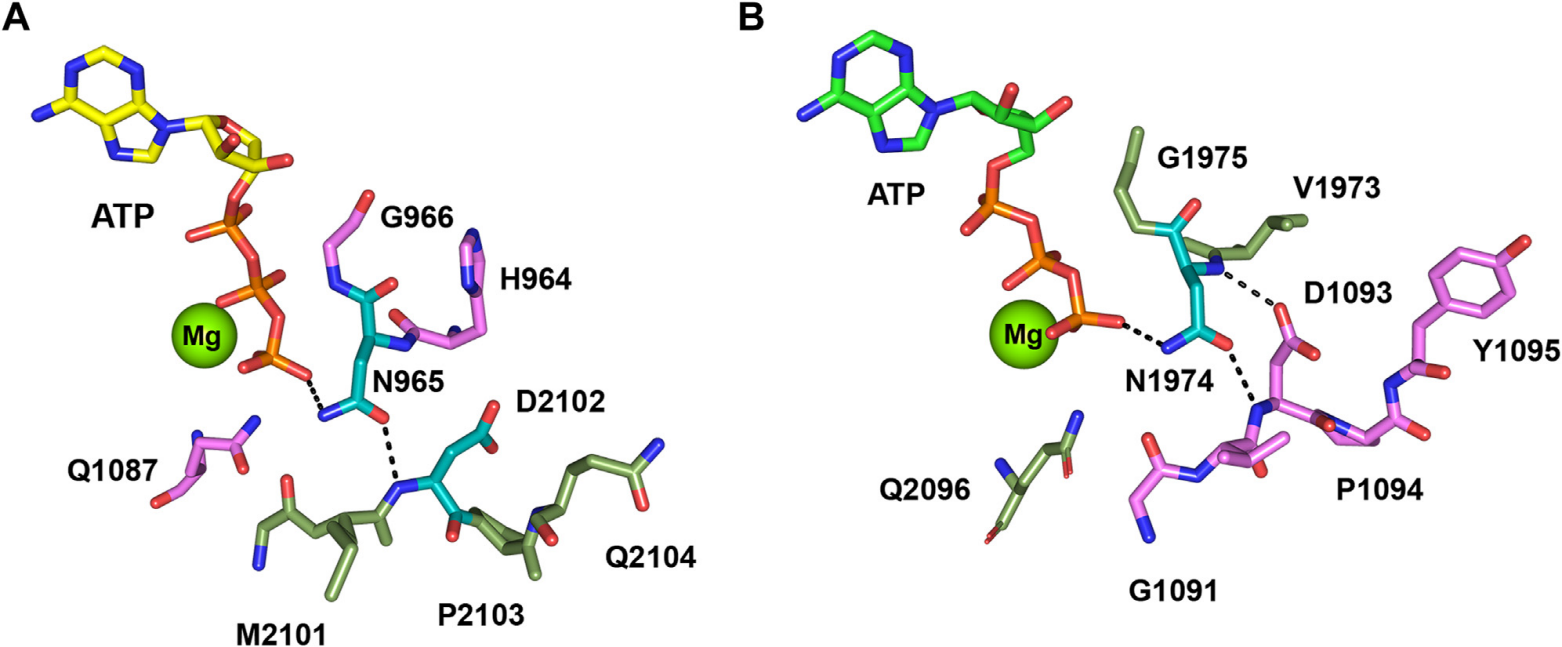
**Polar interactions of the Walker A asparagine residues within ATPase site I and II.***A*, ATPase site 1 showing N965 (*teal backbone*) and adjoining residues (*violet*) of the Walker A motif, Q1087 (E1087 in WT ABCA4) in Walker B, and the opposing D2102 of the D-loop (*teal* backbone) with adjoining residues (*green*). *Dash line* shows likely polar contacts involving the asparagine side chain. *B*, ATPase site 2 showing N1974 (*teal* backbone) and adjoining residues (*green*) of the Walker A motif, Q2096 (E2096 in WT ABCA4), and opposing D1093 of the D-loop (*teal backbone*) and adjoining residues (*violet*). *Dash line* shows polar contacts involving asparagine side chain and asparagine amide with D-loop D1093 aspartate side chain. Asparagine residues come within 2.6 to 3.2 Å to the γ-phosphate of ATP in ABCA4 EQ variant (PDB: 7LKZ) and to the γ-phosphate of AMP-PNP in the ABCA4-AMP-PNP model (PDB: 8F5B) and within 2.8 to 2.9 Å of the main chain amide of the D-loop aspartate residue. Figures derived from the ABCA4 double EQ structure (PDB - 7LKZ). ABC, ATP-binding cassette; PDB, Protein Data Bank

The contribution of residues N965 and N1974 of the Walker A motifs to the structure of the NBDs was assessed from the cryo-EM structures of ABCA4 in its nucleotide-bound state. Both asparagine residues come in close proximity (<3.2 Å) to the gamma phosphate of ATP and additionally appear to form a hydrogen bond with the main chain amide of the aspartic acid residue within the opposing D-loop (D2102 for N965 and D1093 for N1974) (Fig. 6, *A* and *B*). These same polar contacts are also observed in the structure of ABCA4 containing bound AMP-PNP. The low basal ATPase activity and the absence of substrate activated activity for the N965D/K and N1974D/K variants (Fig. 4, *A* and *B*) may result from the incompatibility of these charged residues within the negatively charged environment resulting from the γ-phosphate of ATP, the aspartic acid residue of the D-loop, and the glutamic acid of the Walker B. The tyrosine and to a lesser degree the glutamine substitutions appear to affect the ATPase activity due to steric clashes with surrounding residues within this pocket. In contrast, the smaller neutral residues including serine and alanine can be accommodated within the pocket. However, the loss in polar contacts with the nucleotide and notably the D-loop are likely responsible for the diminished ATPase activities of these variants.

## Discussion

In this study, we determined the structure of ABCA4 containing bound AMP-PNP in each nucleotide-binding site and investigated the role of residues in the NBDs on the functional properties of ABCA4. In the presence of AMP-PNP, ABCA4 displays a closed conformation with the nucleotides wedged between the NBD dimers arranged in a head-to-tail configuration as generally observed for other ABC transporters (32). The α-phosphate of AMP-PNP forms a polar contact with a threonine residue just downstream from the Walker A motif and the β- and γ-phosphate groups interacted with multiple residues within the Walker A motif (Fig. 2). The glutamine of the Q-loop forms polar contacts with phosphate groups as well as the Mg^2+^ ion. Additional contacts are made between the γ-phosphate and serine in the ABC signature motif of the opposing NBD. In this closed conformation, the two TMDs pack together causing the collapse of the *N*-Ret-PE binding site, a finding which is consistent with biochemical studies showing the loss in *N*-Ret-PE binding upon the addition of AMP-PNP (39). Overall, the structure of ABCA4 containing bound AMP-PNP is highly similar to the structure of the ABCA4 EQ double mutant containing bound ATP (27, 29). The conversion from an open outward structure to closed structures upon the binding of ATP has also been reported for the structures of other ABCA transporters including ABCA1 and ABCA7 (37, 38).

To evaluate the role of each NBD in the hydrolysis of ATP, we examined the ATPase activity of ABCA4 variants in which the glutamate residue in each Walker B motif was individually substituted with glutamine. This glutamate residue has been proposed to serve as a general base to polarize a water molecule for hydrolysis of ATP (17, 32). The individual E1087Q and E2096Q variants retain partial basal ATPase activity presumably associated with the ATP site containing the nonmutated Walker B motif since the double EQ mutant is devoid of activity. Although both NBDs contain the structural motifs found in ABC transporters, NBD1 and NBD2 are only 38% identical in sequence. This sequence variation is likely responsible for the differences in the basal ATPase activity observed for the single E-Q variants. Importantly, the *N*-Ret-PE activated ATPase activity is abolished for both the E1087Q and E2096Q variants indicating that binding and hydrolysis of ATP at both ATPase sites are required for the transport cycle. This is supported by the findings that substrate-activated ATPase activity generally correlates with ATP-dependent substrate transport (1, 28). Furthermore, ABCA4 variants with substituents in these glutamate residues (E1087K/D/G and (E2096K/G/V) have been implicated in STGD1. As shown here, the E1087D disease-linked variant, like the E1087Q mutant, is devoid of substrate-activated ATPase activity consistent with the loss in transport function of ABCA4. Evidently, the shorter aspartate side chain cannot substitute for the glutamate residue as a general base to carryout ATP hydrolysis. Reduced basal ATPase activity and the absence of substrate-activated activity have also been previously reported for the E1087K and E2096K variants (13, 42). At a clinical level, an individual homozygous for the E1087K mutation had a severe form of STGD1 similar to that observed for patients with null mutations in both ABCA4 alleles (44). This result is consistent with the complete loss of ABCA4 transport activity of this E1087K variant. Taken together, these results support the view that ATP hydrolysis at both NBDs is required for the function of ABCA4 as an ATP-dependent transporter.

The Walker A motif is known to play a crucial role in the binding of nucleotides to ABC transporters with multiple residues contacting the β,γ phosphate residues of ATP (Fig. 2). The N965 and N1974 residues in the respective NBD Walker A motifs of ABCA4 are of particular interest since various missense mutations in these residues have been implicated in STGD1. In this study, we have confirmed the importance of these asparagine residues using site-directed mutagenesis together with functional activity measurements. Substitution of either asparagine with other residues diminishes, and in many cases, abolishes substrate-dependent activity of ABCA4. Structural studies of ABCA4 in its ATP and AMP-PNP bound states show that the asparagine side chain of each Walker A motif forms polar contacts with the γ-phosphate of the nucleotide and hydrogen bond with the main chain amide of the aspartate of the opposing NBD D-loop (Fig. 6). Similar contacts between the Walker A asparagine residue and the nucleotide and D-loop were previously reported for the structure of the bacteriophage T4 ABC protein, Rad50 (45). This finding and activity measurements led to the proposal that the asparagine residues play a crucial role in increasing the affinity for ATP and orienting the γ-phosphate for nucleophilic attack by the catalytic water molecule. Our studies are consistent with this proposal. As in the case of other ABC transporters, the D-loop is present at the NBD dimer interface but unlike the signature motif it does not directly interact with the nucleotide. In the nucleotide-bound structure of ABCA4, the aspartate side chain of the NBD1 D-loop (D1093) appears to form a hydrogen bond with main chain amide of the NBD2 Walker A asparagine (N1974), an interaction also observed for the bacteriophage T4 Rad50 (45). However, D2022 of NBD2 which extends into the pocket containing the N965 residue of NBD1 is further away and therefore may not interact with the main chain amide of the N965 residue. It is interesting to note that mutations in these aspartate residues (D1093N/A/G/E and D2022/A/G/E) have been implicated in STGD1 indicating their importance in the structure and function of ABCA4. The detailed mechanism for the role of the D-loop aspartate residues, however, remains to be determined.

In the case of the N965S mutation, there is modest substrate-activated ATPase activity when isolated from either transfected HEK293 cells or transgenic mice (Figs. 4 and 5). This residual activity likely explains the moderate-severe phenotype of individuals homozygous for this mutation. On the other hand, substitution of either of these Walker A asparagine residues with amino acids containing charged or bulky side chains results in a complete loss in substrate-activated activity. Individuals homozygous for these mutations or when paired with a null allele would be predicted to have a severe form of STGD1 characterized by an early onset and rapid disease progression as a result of complete loss in ABCA4 functional activity.

Although the present study has focused on the structural and functional characterization of mutations within the Walker A and Walker B motifs, over 350 different missense mutations within the NBDs of ABCA4 have been implicated in STGD1 (https://databases.lovd.nl/shared/variants/ABCA4/). A few of these have been analyzed at a functional level, but their involvement in the structure of ABCA4 has not been analyzed. With the structures of ABCA4 in various states available, it is now possible to gain further insight into the mechanisms by which substitutions in key residues can cause a loss in function of ABCA4 and STGD1. Several residues in the NBDs associated with STGD1 (Fig. 1*C*) are discussed below.

**Figure 7:**
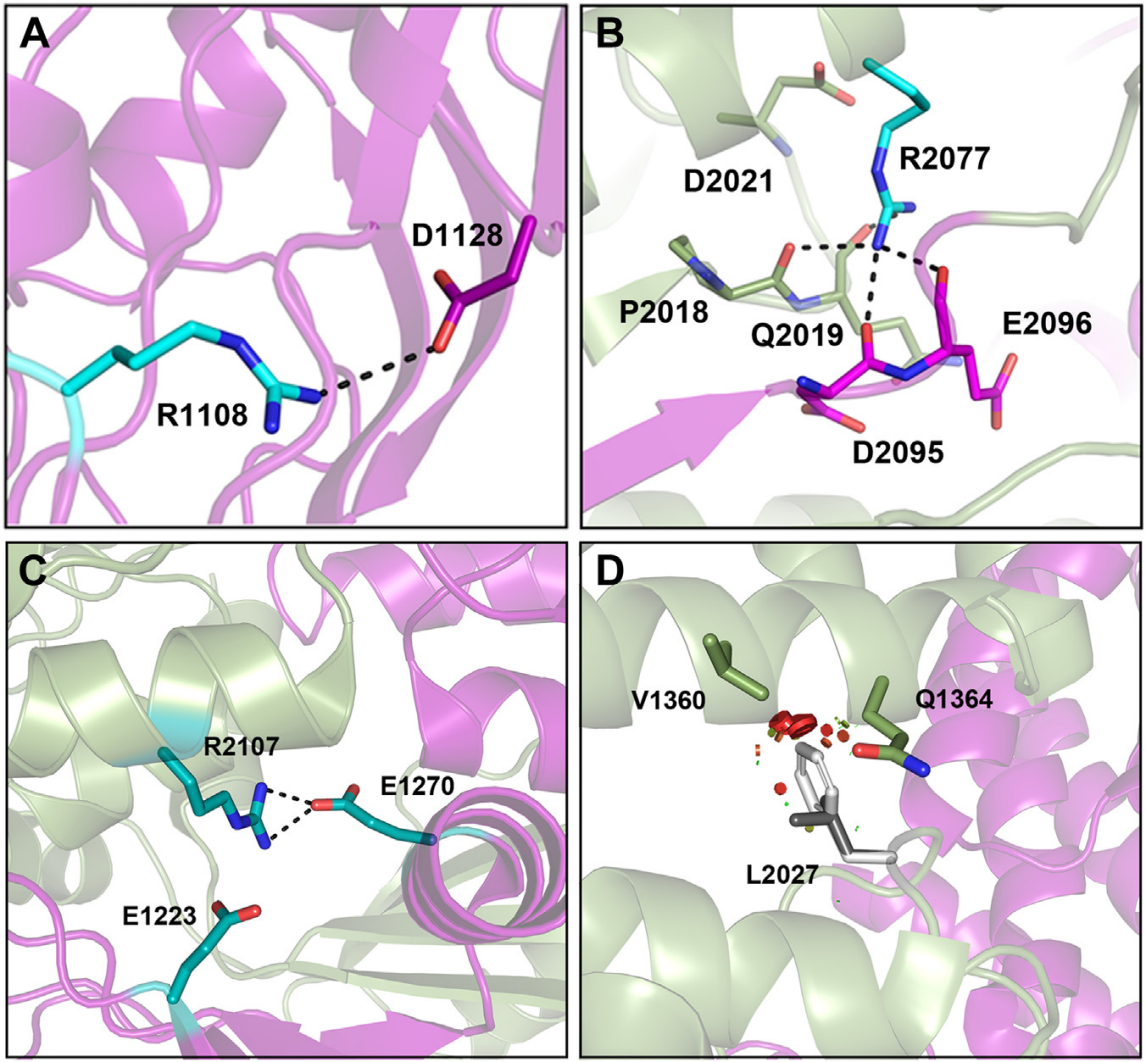
**Structure of NBDs highlighting interactions involving several residues when mutated are known to cause Stargardt disease (STGD1).***A*, electrostatic interaction of R1108 side chain with D1128 side chain within NBD1 (*purple*). (ABCA4 PDB: 8F5B). Interaction is lost in R1108C STGD1 variant. *B*, hydrogen bonds between the R2077 side chain and with main chain carbonyl residues of D2095 and E2096 of the Walker B motif (*magenta*) and P2018 and Q2019 of the Q-loop of NBD2 (*green*). (PDB: 8F5B). Interactions are lost in the R2077W STGD1 variant. *C*, electrostatic interaction between R2107 side chain in NBD2 (*green*) with the side chains of E1223 in RD1 and E1270 in pinning helix (PH1) of NBD1 (*purple*) in nucleotide-bound state. (PDB: 7LKZ). Some of the interactions are retained in the R2107H STGD1 variant resulting in retention in significant activity. *D*, the location of the L2027 residue within NBD2. This residue is within a pocket bordering the IH3 helix suggested to be important in coupling ATPase activity with transport. Substitution of this leucine (*dark gray*) with phenylalanine (*light gray*) in the L2027F STGD1 variant causes a significant clash with IH3 (*red discs*) as analyzed Pymol. (PDB: 7LKP). ABC, ATP-binding cassette; IH3, intracellular helix 3; NBD, nucleotide-binding domain; PDB, Protein Data Bank; RD, regulatory domain; STGD1, Stargardt disease.

The R1108C variant in NBD1 has been linked to a moderate-to severe form of STGD1 (44). At a biochemical level, this variant displays a reduced level of expression and diminished basal and substrate-activated ATPase activity (13). In the structures of ABCA4 in the presence and absence of nucleotide including the structure containing bound AMP-PNP, R1108 located at the end of an α-helix forms a salt bridge with the side chain carboxyl group of D1128 located within a loop joining β-strands that stabilize the Walker A motif of NBD1 (Fig. 7*A*). The loss in the positively charged side chain and corresponding ionic interaction with D1128 likely destabilizes the overall structure of ABCA4 resulting in the observed reduction in expression and diminished hydrolysis of ATP. Interestingly, a mutation in D1128 (D1128G) has also been associated with STGD1, but this variant has not been evaluated at a biochemical level.

The R2077W mutation in NBD2 is associated with a severe, early onset form of STGD1 when paired with a null allele (46). At a biochemical level, this mutation results in a significant reduction in both protein expression and basal ATPase activity and complete loss in substrate-mediated ATPase activity and *N*-Ret-PE binding (47). In structural models, the side chain of R2077 extends from an α-helix into a pocket surrounded by segments from the Q-loop, the Walker B motif, and the aspartate side chain of D2021 (Fig. 7*B*). In the nucleotide-free state, the R2077 comes within hydrogen bonding distance to the main chain carbonyls of Q2019 and P2018 as well as the main chain carbonyls of E2096 and P2097. These polar interactions appear to be crucial for the proper folding and stability of ABCA4 into a functional transporter. Loss of these interactions together with considerable steric clashes of tryptophan with residues within the pocket are likely responsible for severe protein misfolding as indicated by the decrease in expression levels and lack of functional activity.

Although a large number of missense mutations in ABCA4 are known to cause early onset, severe STGD1, some mutations produce a late-onset, milder disease. An example is the R2017H mutation present at relatively high frequency in the African-American population (48). In particular, an individual homozygous for this mutation was diagnosed with only mild vision loss in his seventh decade of life. Biochemical studies indicate that the R2107H variant expresses at close to WT levels and displays significant basal and substrate-activated ATPase activity consistent with the mild phenotype of the STGD1 patient (13). At a structural level, R2107 extends from a helical segment of NBD2 into a space bordering RD1 and the downstream pinning helix 1 (27, 29). In the nucleotide-bound state, the R2107 side chain forms a salt bridge with E1270 in pinning helix 1 and is in close contact with E1223 in RD1 (Fig. 7*C*). This interaction appears to contribute to the stability of the NBD2 and its ATPase activity. The positively charged histidine residue can partially substitute for arginine resulting in only a modest decrease in the expression and activity of ABCA4. However, other disease-associated mutations such as the R2107P would likely have a more detrimental effect on the structure and activity through the disruption of the helical segment by the proline residue and complete loss in the ionic interactions. As a result, one would predict that such a variant would result in a severe form of STGD1.

A number of mutations involve residues that do not involve polar side-chain interactions. An example is the L2027F mutation in NBD2 of ABCA4. Individuals homozygous for this variant typically display a moderate phenotype with an age of onset typically within the third decade of life (49). The L2027F variant exhibits a reduction in expression (∼40% WT levels) presumably due to considerable protein misfolding, but retains some substrate-dependent activity (13). Within the structure of ABCA4, the L2027 residue extends from a helical segment of NBD2 toward the intracellular helix 3 that is present just prior to transmembrane segment 7 within the TMD2 of ABCA4 (Fig. 7*D*). These intracellular helices generally couple conformational changes within the NBDs to conformational changes in the TMDs. Substitution of the leucine 2027 with phenylalanine results in considerable steric clashes with intracellular helix 3 (Fig. 7*D*).

In summary, our studies indicate that ATP hydrolysis at both NBDs is required for the function of ABCA4 as an *N*-Ret-PE transporter. The structures of ABCA4 in various states coupled with protein expression/stability and functional characterization provides mechanistic insight into the function of ABCA4 and the pathogenesis of STGD1.

## Experimental Procedures

### Reagents

The human ABCA4 complementary DNA (NCBI: NP_000341.2) engineered to contain a C-terminal 1D4 tag and cloned into pCEP4 has been described previously (50). Missense mutations were generated by PCR based site-directed mutagenesis as previously described (47). All DNA constructs were verified by Sanger DNA sequencing. Monoclonal antibody Rho1D4, originally generated in house (51), was obtained in bulk from University of British Columbia (https://ubc.flintbox.com/technologies/0f1ef64b-fa5d-4a58-9003-3e01f6f672a6); the Rim 3F4 antibody has been described previously (3). These antibodies were coupled to CNBr-activated Sepharose 4B as previously described (51). Phospholipids including 1,2-dioleoyl-sn-glycero-3-phosphoethanolamine (DOPE), and brain polar lipids (BPLs) were obtained from Avanti Polar lipids. ATP, AMP-PNP, and ATR were purchased from MilliporeSigma and CHAPS and glyco-diosgenin (GDN) were obtained from Anatrace.

### Expression and purification of ABCA4

HEK293F suspension cells were transfected with plasmid DNA using polyethylenimine MAX (Polysciences) as previously described (28). After 72 h, the cells were harvested and frozen at −80 °C until required. ABCA4 was purified as previously described (28). Briefly, 10 g of cells were thawed and resuspended in resuspension buffer (25 mM Hepes, pH 7.4, 150 mM NaCl, 5 mM MgCl_2_, and 1 mM DTT) containing benzonase (Sigma-Aldrich) and protease inhibitor (1:1000) (Millipore). The crude cell lysate was added dropwise to the solubilization buffer (25 mM Hepes, pH 7.4, 150 mM NaCl, 5 mM MgCl_2_, 1 mM DTT, 0.01 mg/ml DOPE, and 18 mM CHAPS) containing the protease inhibitor and stirred at 4 °C for 2 h. The solution was centrifuged at 64,000*g* for 60 min in a Beckman SW28 rotor to remove any aggregated material. The supernatant was then added to a Rho1D4 immunoaffinity matrix at 4 °C for 1.5 h with gentle mixing. The matrix was then washed with 15 volumes of size-exclusion chromatography buffer (25 mM Hepes, pH 7.4, 150 mM NaCl, 5 mM MgCl_2_, 1 mM DTT, 0.01 mg/ml DOPE, 10 mM CHAPS) and subsequently washed with size-exclusion chromatography buffer consisting of 25 mM Hepes, pH 7.4, 150 mM NaCl, 5 mM MgCl_2_, 1 mM DTT, 0.01 mg/ml DOPE, and 0.04% GDN) to exchange CHAPS with GDN. ABCA4 was eluted in the same buffer containing the 0.5 mg/ml 1D4 peptide at 18 °C and subsequently separated from aggregated protein on a Superose 6 column (Cytiva).

The expression of WT and ABCA4 variants were quantified on Coomassie Blue stained gels or Western blots labeled with the Rho1D4 or Rim3F4 monoclonal antibody using a LiCor Odyssey imager and software (https://www.licor.com/bio/image-studio-lite/ ) as previously described (21, 43).

### Sample preparation for single-particle cryo-EM and data collection

The sample of purified ABCA4 was concentrated to 4.3 mg/ml and incubated with 2 mM AMP-PNP for 40 min at 23 °C. A 3 μl aliquot of the ABCA4.AMP-PNP complex was applied on plasma cleaned UltrAUfoil R1.2/1.3300 mesh (Electron Microscopy Science). Grids were blotted for 2 s with a blotting force of −8 and 100% humidity and plunged into liquid ethane using the Vitrobot Mark IV (Thermo Fisher Scientific) located in the high-resolution macromolecular cryo-electron microscopy at UBC. Grids were screened at high-resolution macromolecular cryo-electron microscopy in a Glacios EM (Thermo Fisher Scientific) at 200 kV equipped with a Falcon 3 (Thermo Fisher Scientific) direct detector and selected grids were used for data collection at Pacific Northwest Cryo-EM center (PNCC–Oregon/US).

Electron micrographs were acquired with a Titan Krios G3i at 300 kV equipped with Gif K3 (Gatan) direct detector at 81,000×. Movies were collected with SerialEM using a super-resolution mode, with a nominal pixel size of 0.5395 Å/pix, a stack of 50 frames, and a total dose of 50 e−/Å2. A total 8,091 movies were collected for the nucleotide-bound complex with a defocus range from −0.8 μm to −2.2 μm.

### Single-particle cryo-EM data processing

Patch motion correction and contrast transfer function correction were performed with cryoSPARC v.3.012 (https://cryosparc.com/) (52). Subsequently, 3,075 particles were manually picked for initial template generation that was used in template picker. A template-free particle picking was also performed to improve the final number of particles, which resulted in total 825,604 particles that were used in 3D classification. Duplicated particles were removed and particles belonging to the representative group were submitted for another round of 2D classification followed by nonuniform refinement, 13 with 149,336 particles. A final “gold standard” resolution of 3.9 Å was achieved for the nucleotide bound complex (Fig. S5).

### Model building

The ABCA4 EQ bound to ATP structure (Protein Data Bank ID 7LKZ) (27) was used as a starting model for WT ABCA4 bound to AMP-PNP. First, the full-length ABCA4-EQ was docked into the map using the UCSF Chimera package from the Resource for Biocomputing, Visualization, and Informatics at the University of California, San Francisco (supported by NIH P41 RR-01081) (53). The model was iteratively refined using Coot v0.9 (54) (https://www2.mrc-lmb.cam.ac.uk/personal/pemsley/coot/) and real-space refinement under Phenix v.1.19 to 4092 environment (https://phenix-online.org) (55, 56). ABCA4.AMP-PNP structure was refined and validated (Table S1). It contains 10 sugar molecules and 1924 amino acids. Figures were generated in ChimeraX1.4 (53) or Pymol Molecular Graphics System (version 1.7 Schrodinger, LLC).

### Immunofluorescence microscopy

Immunofluorescence microscopy was performed as previously described (43). Briefly, COS-7 cells grown on coverslips coated with poly-L-lysine were transfected with 1μg of plasmid and 3 μg of polyethylenimine. At 48 h post transfection, cells were fixed with 4% paraformaldehyde in 0.1M phosphate buffer (PB), pH 7.4, for 25 min and subsequently washed in PBS. The cells were blocked with 10% goat serum, 0.1% Triton X-100, and PB for 30 min. The cells were then labeled for 2 h with the hybridoma culture fluid containing the Rho1D4 monoclonal antibody (1:50 dilution) to detect ABCA4 and calnexin rabbit polyclonal antibody as an endoplasmic reticulum marker. The coverslips were washed in PB and subsequently labeled with secondary antibodies (Alexa-488 goat-anti-mouse Ig and Alexa-594 goat anti-rabbit Ig) and counterstained with the nuclei stain (4′,6-diamidino-2-phenylindole) for 1 h. The coverslips were subsequently washed, mounted onto microscope slides, and visualized under a Zeiss LSM700 confocal microscope using a 40X objective lens. Images were analyzed using Zeiss Zen software (https://www.zeiss.com/microscopy/en/products/software/zeiss-zen-lite.html).

### ATPase assay and *N*-Ret-PE binding assay

ATPase assays were carried out on immunoaffinity purified ABCA4-1D4 variants from HEK293 transfected cells as previously described (28, 43). ATP concentration was typically 1 mM or as described in the figures. Solid-phase *N*-Ret-PE binding assays in the absence and presence of 2 mM ATP was performed as previously described using tritiated ATR at a specific activity of 500 dpm/pmol (43).

### ATPase assay on mouse retinal photoreceptor membranes

Retinal membranes from age-matched WT, homozygous, and heterozygous N965S ABCA4 transgenic mice, and ABCA4 KO mice were isolated as previously described (21). For ATPase activity measurements, membranes from 28 eyes of 2-month-old N965S mice or 14 eyes of 2-month-old WT or ABCA4 KO mice were solubilized for 1 h in CHAPS buffer (18 mM CHAPS detergent, 10 mM Hepes, 1 mM DTT, 5 mM MgCl_2_, 0.2 mg/ml BPL, and 0.002% cholesteryl hemisuccinate (Avanti Polar Lipids)). After centrifugation at 40,000g for 20 min to remove any aggregated material, the supernatant was applied to a Rim3F4-Sepharose 2B immunoaffinity column with gentle mixing for 1 h at 4 °C. After extensive washing in column buffer containing 10 mM CHAPS, 10 mM Hepes, 1 mM DTT, 5 mM MgCl_2_, 0.2 mg/ml BPL, and 0.002% cholesteryl hemisuccinate to remove unbound protein, the ABCA4 protein was eluted with column buffer containing 0.2 mg/ml 3F4 peptide and directly used for ATPase activity assays. Briefly, 18 μl of protein was incubated with 0.1 mM ATP with or without 40 μM ATR for 30 min at 37 °C in a total volume of 20 μl. The reaction was stopped by the addition of an equal volume of ADP-Glo reagent (Promega) and ATP/ADP was measured as per manufacturer guidelines. Each sample was analyzed in triplicate. Three independent experiments were carried out using the same amount of ABCA4 protein except for the ABCA4 KO mice. Protein concentration was determined on Coomassie blue stained SDS gels using bovine serum albumin as a standard.

### Statistical Analysis

Statistical analysis of the data was carried out using GraphPad Prism 9.0. (https://www.graphpad.com/) Data were expressed as a mean ± SD for n ≥ 3. When applicable unpaired *t* test was used.

## Data availability

The cryo-EM density map has been deposited in the Electron Microscopy Data Bank under accession codes: EMD-28864 and the coordinates have been deposited in the Protein Data Bank under the accession code: PDB ID 8F5B.

## Supporting information

This article contains supporting Information (58).

## Conflict of interest

The authors declare that they have no conflicts of interest with the contents of this article.
