# Supplementary material for: Structural and functional characterization of the nucleotide-binding domains of ABCA4 and their role in Stargardt disease
Source: J Biol Chem. 2024 Aug 14;300(9):107666. doi: 10.1016/j.jbc.2024.107666 (PMC11405800; doi:10.1016/j.jbc.2024.107666)
Supplement: Supporting information [file mmc1.docx]

**Supporting Information**


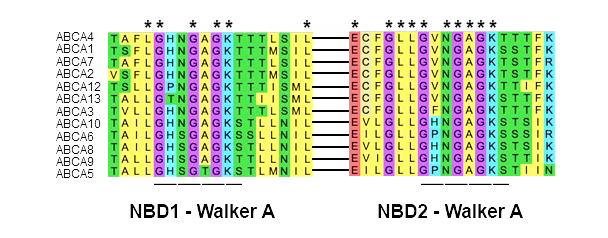


**Figure S1**: Alignment of ABCA segments containing the Walker A motifs (dashed lines) for NBD1 and NBD2. There is a high degree of conservation for the asparagine (N) residue within the Walker A motifs. * most highly conserved residues.


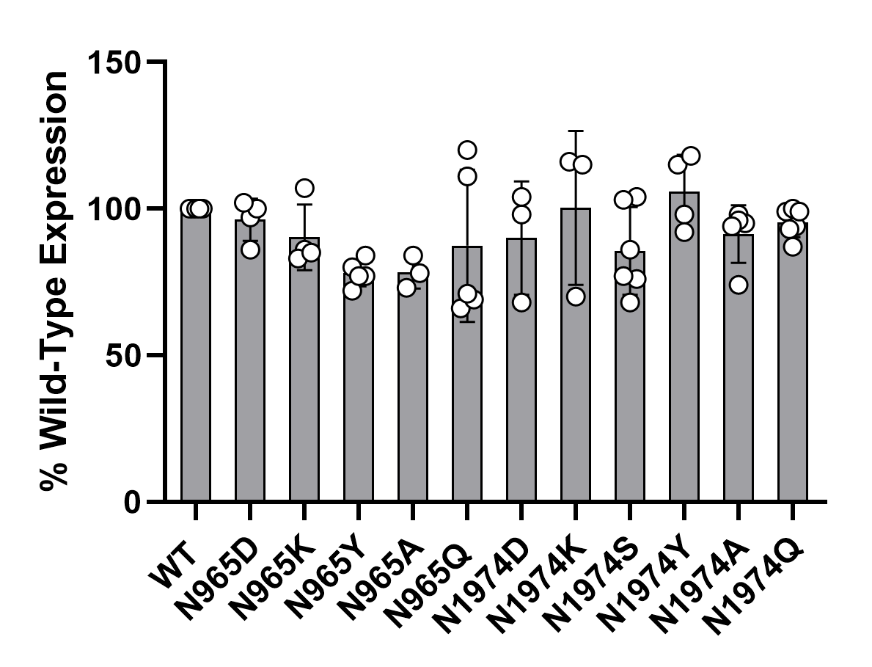


**Figure S2:** Expression levels of ABCA4 variants containing substitutions in N965 and N1974 as determined by Western blotting. HEK293T cells were transfected with the ABCA4 variants. The cell lysates were solubilized in CHAPS detergent, centrifuged to remove any aggregated material. The supernatant (7 µg protein) was applied to each lane for analysis by SDS gel electrophoresis followed by Western blotting. All HEK293 variants express at close to WT ABCA4 levels. Data points (circles) reflect independent experiments. Bars indicate SD.


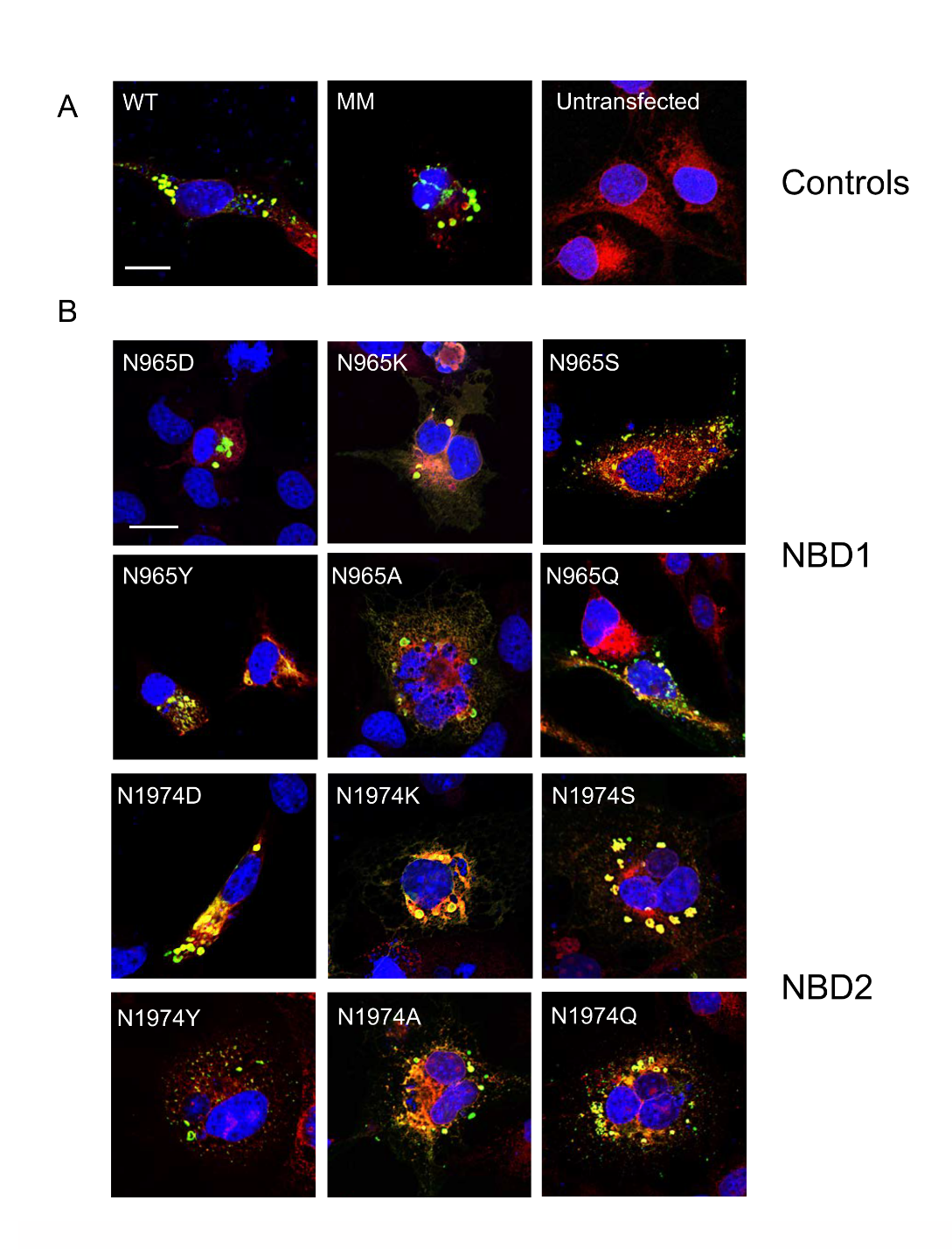


**Figure S3**: Representative immunofluorescence micrographs of ABCA4 variants containing substitutions in the N965 and N1974 residues expressed in culture cells. All variants showed the presence of vesicular like intracellular structures with some reticular staining. ABCA4 variants are stained in green, endoplasmic reticulum marker is stained in red, and nuclei are stained with DAPI nuclear stain (blue). WT represents wild-type ABCA4 and the MM control is the variant in which the Walker A lysine residues were replaced with methionine. Scale Bar: 10µm.





**Figure S4**: Representative SDS gels of purified ABCA4 variants containing substitutions in the N965 and N1974 residues of NBD1 and NBD2, respectively. WT or ABCA4 variants were expressed in HEK293T cells. The cell lysates were solubilized in CHAPS detergent and purified on a Rim3F4 immunoaffinity column. SDS gels were stained with Coomassie Blue. MM is an ABCA4 variant in which the lysine residues within the Walker A motifs were substituted with methionine residues. Note - Two lanes contain N965A to show reproducibility of protein loading. Data for N965S purification have been published previously (1).

1. Molday, L.L., Djajadi, H., Yan, P., Szczygiel, L., Boye, S.L., Chiodo, V.A., Gregory-Evans, K., Sarunic, M.V., Hauswirth, W.W. and Molday, R.S. (2013) RD3 gene delivery restores guanylate cyclase localization and rescues photoreceptors in the Rd3 mouse model of Leber congenital amaurosis 12. *Hum. Mol. Genet*., **22**, 3894–3905


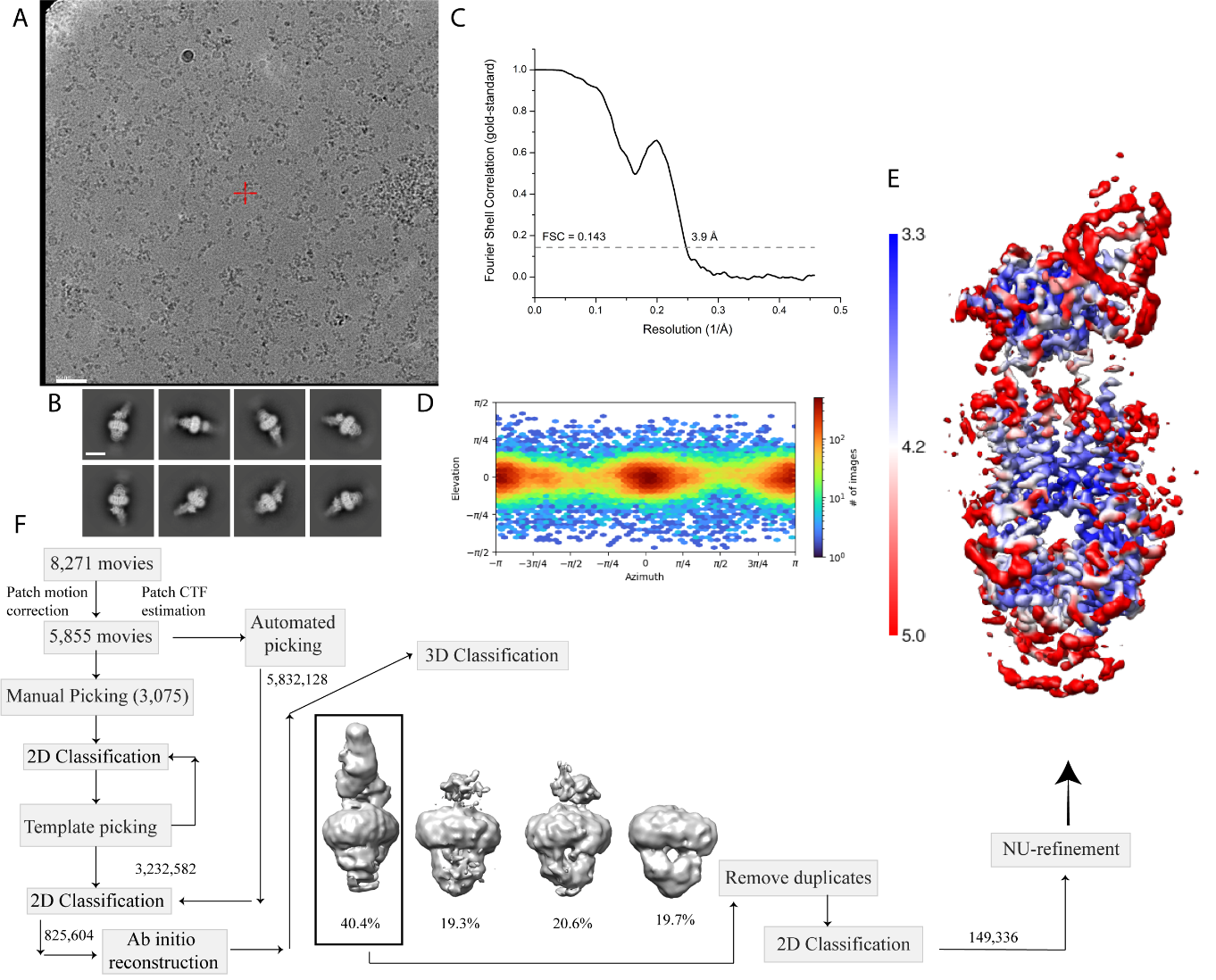


**Figure S5**. Cryo-EM of ABCA4 containing AMP-PNP. **(A)** Representative cryo-EM micrograph. The micrograph is a representative of the 8,271cryo-EM images Bar: 50nm. **(B)** Representative 2D class averages. Bar: 10nm. **(C)** Gold standard Fourier Shell Correlation (FSC) indicates a final resolution of 3.9 Å. **(D)** Azimuth plot of angular distribution shows lack of one orientation (top view). (**E**) Local resolution map for ABCA4.AMP-PNP calculated in cryoSPARC v.3.0 **(F)** Flowchart for the cryoEM data processing using cryoSPARC v3.0.

**Supplemental Table S1**

| **Data collection and processing** | **ABCA4.AMP-PNP** |
| --- | --- |
| Microscope | Titan Krios G3i |
| Voltage (kV) | 300 |
| Detector | K3 |
| Pixel size (Å/pixel) | 0.5395 |
| Defocus range (µm) | -0.8 / -2.2 |
| Movies | 8,271 |
| Frames per movie | 50 |
| Electron exposure (e^-^/Å^2^) | 50 |
| Initial number of particles (3D classification) | 825,604 |
| **Refinement** |  |
| Symmetry | C1 |
| Final number of particles | 149,336 |
| Resolution (Å)  FSC threshold 0.143 | 3.95 |
| Sharpening factor | Local sharpening - automatic |
| **Model composition** |  |
| Non-hydrogen atoms | 14434 |
| Protein residues | 1810 |
| Sugar molecules | 7 |
| **Bonds R.M.S. Deviations** |  |
| Length (Å) | 0.005 |
| Angles (º) | 1.018 |
| **Validation** |  |
| Molprobity Score | 2.17 |
| Clash score | 8.63 |
| Favored rotamer (%) | 98 |
| **Ramachandran plot (%)** |  |
| Favored | 92.67 |
| Allowed | 7.33 |
| Outlier | 0.00 |
| **Data availability (PDB ID)** |  |
| **PDB ID** | 8F5B |
| **EMD** | 28864 |
